# Health risk assessment of heavy metal pollution and its sources in agricultural soils near Hongfeng Lake in the mining area of Guizhou Province, China

**DOI:** 10.3389/fpubh.2023.1276925

**Published:** 2023-11-10

**Authors:** Wengang Cui, Yan Mei, Suihua Liu, Xinding Zhang

**Affiliations:** ^1^College of Geography and Environmental Sciences/College of Karst Science, Guizhou Normal University, Guiyang, Guizhou, China; ^2^Key Laboratory of Mountainous Resources and Environmental Remote Sensing Applications, Guiyang, Guizhou Normal University, Guiyang, Guizhou, China

**Keywords:** heavy metal pollution, APCS-MLR receptor model, human health risks, Hongfeng Lake, agricultural soil

## Abstract

**Background:**

Accelerated modern industrial processes, extensive use of pesticides and fertilizers and remaining issues of wastewater irrigation have led to an increasingly severe composite pollution of heavy metals in arable land. Soil contamination can cause significant damage to ecological environments and human health. Mineral resource mining can result in varying degrees of heavy metal pollution in surrounding water systems and soil. As a plateau lake, Hongfeng Lake has a fragile watershed ecosystem. Coupled with the rapid development of the current socio-economy and the ongoing activities of mining, urbanization and agricultural development, the water and soil environment of the lake and arable land are facing serious heavy metal pollution. Therefore, the situation warrants attention.

**Methods:**

This study focused on characterizing soil types and conducted sampling and laboratory testing on the farmland soil in Hongfeng Lake. The integrated Nemero comprehensive pollution assessment and potential ecological pollution assessment methods were used to evaluate the heavy metal pollution status. The APCS-MLR model was employed to explore the sources of heavy metal pollution. In addition, the human health risk model was used to analyze the association between heavy metal content in cultivated land and human health risks.

**Results:**

The single-factor pollution of each element was ranked in descending order: Hg > As > Pb > Cr > Cd, with Hg being the main pollutant factor. The entire area was subjected to mild pollution according to the pollution index. Pollution source analysis indicated two main pollution sources. Hg, As, Pb and Cr pollution mainly resulted from Source 1 (industrial and natural activities), accounting for 71.99%, 51.57%, 67.39% and 68.36%, respectively. Cd pollution was mainly attributed to Source 2 (agricultural pollution source), contributing 84.12%. The health risk assessment model shows that heavy metals posed acceptable carcinogenic risks to humans rather than non-carcinogenic risks. As was the main non-carcinogenic risk factor, while Cr was the main carcinogenic risk factor, with higher risks in children than adults.

**Conclusion:**

Our study identified the heavy metal pollution in farmland soil in Hongfeng Lake, evaluated and analyzed the pollution sources and identified the heavy metal elements in cultivated lands that have the greatest impact on human health risks. The aim of this study is to provide a scientific basis for soil heavy metal pollution control.

## 1. Introduction

Accelerated modern industrialization, extensive pesticide and fertilizer use and longstanding issues of sewage irrigation have led to increasingly severe heavy metal complex pollution in agricultural soils (1–4). Mining activities can also cause varying levels of heavy metal pollution in water systems and agricultural soils. For example, research on a copper mine in the Jiulong section of the Yalong River basin found that the Cu element was more likely to accumulate and cause pollution in some upstream water systems. These systems were closer to the mining point, at higher elevations, in steeper terrain and with smaller water convergence (5). In addition, it has also been found that heavy metal contents in farmland soils surrounding the Nhue River mining area in Vietnam far exceeded background values, and Cd content exceeded the heavy metal standard for Vietnamese agricultural soils (6). Fry found that soils at a copper smelter in Namibia were contaminated with As, Cu, and Pb, and surface dust on buildings posed a health risk to humans (7). Therefore, it is necessary to assess heavy metal pollution in soils surrounding mining areas in watersheds in order to address pollution-induced risks.

This study took the Hongfeng Lake basin as the study area. Hongfeng Lake was formed in 1958 as an artificial lake to construct the Maotiao River cascade hydropower station with fragile watershed ecosystems. It is the largest source of drinking water in Guiyang City. With the rapid development of the current socio-economy, mining, urbanization construction and agricultural development activities, the water environment of the lake is in a critical situation. Hongfeng Lake is mainly fed by the Maiweng River, the Yangchang River, the Maxian River, and the Houshui River. There are many factories and enterprises in the upstream watershed, such as chemical, metallurgical and mining enterprises. The watershed has been receiving industrial and mining wastewater for a long time, and the watershed soils have been polluted to a certain extent (8, 9).Soil heavy metals can migrate to aquatic ecosystems, threatening the drinking water of watersheds. Since soil environmental conditions interact with water quality in watersheds, soils in watersheds serve as a region of aquatic ecosystems. Excessive soil heavy metals can inhibit soil functions, alter soil physicochemical properties, disrupt nutrient supply and balance and impact the ecological environment and crop growth (10–12). This can threaten crop growth and development, agricultural production security and human health through various exposure pathways during human labor. For the sake of human society, life and ecology, it is vital to evaluate and explore the soil pollution status and sources in the Hongfeng Lake basin. We should pay attention to the agricultural soil safety in the Hongfeng Lake basin. Studies should focus on refining soil types and selecting cultivated land soils that have the most significant impact on ecosystems and human health in the watershed. The pollution levels and sources of the five heavy metals (Hg, As, Pb, Cr, and Cd) in the cropland soil of the watershed should also be clarified.

Heavy metal pollution sources have been widely explored using receptor and diffusion models. The diffusion models require information on discharge data and related information of each pollution source to predict the spatiotemporal changes of individual pollutants. The receptor models require the chemical content and related microscopic analysis of each collected sample. Establishing the corresponding relationship between pollutant sources and pollution status can help to identify the main pollution sources and their contributions to the receptors (13). The receptor models are more widely used with fewer restrictions, mainly including the chemical mass balance model (CMB), principal component analysis (PCA) and positive matrix factorization (PMF) models (14, 15). In particular, the absolute principal component score combined with multiple linear regression analysis (APCS-MLR) focuses on receptor pollution sources. This technique does not require knowledge of the number of pollution sources and can easily and objectively determine the specific contribution of pollution sources to the receptors with simple operating conditions (16, 17). The method first proposed by Thurston and Spengler in 1985 focuses on obtaining APCS of factors through factor analysis and then calculating the contribution rate of common factors to pollutants using APCS-MLR (18). This model has been widely used to analyze pollution sources in air, surface water and sediment (19, 20).

Therefore, this study refined the soil types and selected cropland soils affecting ecology and human health the most as the research object. The pollution level of five soil heavy metals in the cropland of the watershed was evaluated. The APCS-MLR receptor model was used to analyze soil heavy metal sources. Combined with the spatial distribution and correlation analysis of heavy metal pollution, pollution levels, sources and distribution characteristics of heavy metals in the cropland in the mining area of the Hongfeng Lake basin were clarified. The human health risk impact assessment method defined by the US Environmental Protection Agency (USEPA) was used to evaluate the health risks of heavy metals to humans. This study can provide a reference and scientific basis for preventing and controllinh farmland heavy metal pollution and health risks in Hongfeng Lake.

## 2. Materials and methods

### 2.1. Study area

The Hongfeng Lake basin is located in the suburbs of Qingzhen City with more than 30 types of mineral deposits. These deposites include bauxite, hematite, pyrite, coal and marble. Bauxite is one of the primary mineral sources of Guizhou Aluminum Corporation. Hongfeng Lake is mainly fed by the Maiweng River, the Yangchang River, the Maxian River and the Houshui River. The reservoir has an area of 57.2 km^2^, a water level of about 1,240 m, a dead water level elevation of 1,127.5 m and a capacity of 752.9 million m^3^. There are 67 water supply points. Hongfeng Lake supplies 250,000 tons of water daily to Guiyang City.

### 2.2. Sampling, processing, and analysis

**Figure 1:**
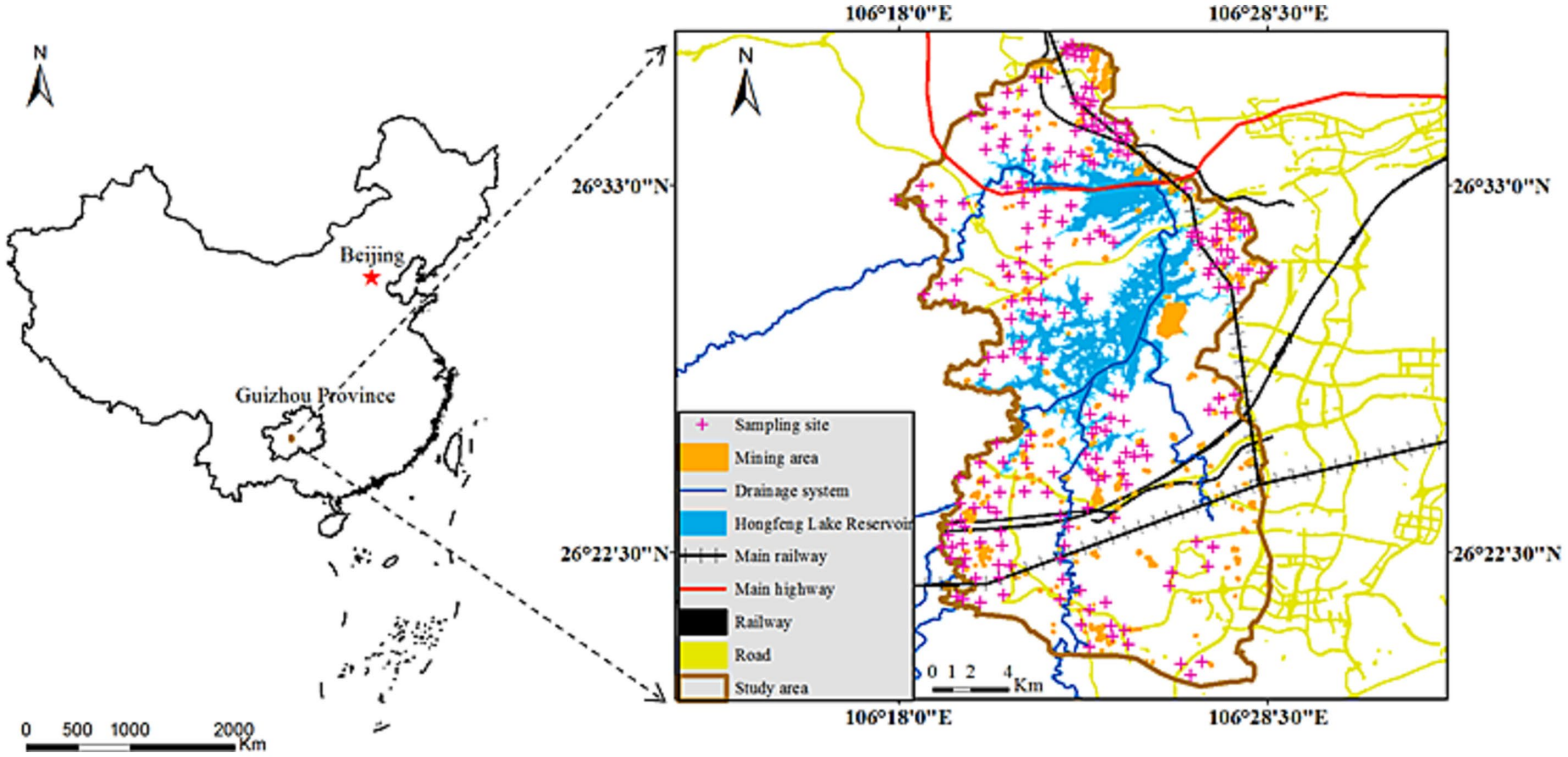
Schematic diagram of sampling points.

The sampling point is shown in Figure 1. From August to December 2020, after preliminary investigations, sampling points were arranged radially from Shangjian Street in the upstream station of Hongfeng Lake to Hongfeng Town, Gaofeng Town and Machang Town downstream. The sampling points were set up based on the geographical location and climatic conditions of the mining area. These points were in the farmland in the vicinity of the mine, tailings and beneficiation plants using the plum blossom pattern. Individual soil points consisting of 5 sub-samples were taken at 20 cm using a small shovel. A total of 190 soil samples were collected. Next, the soil samples were placed in a polyethylene bag, crushed and sealed into bottles for subsequent analysis. Heavy metals were determined primarily using reagent blanks and national standard soil samples (GSS-3). One sample was randomly selected from every 30 samples for triple parallel analysis to ensure quality control. Cd, As, Pb and Cr contents were measured using an ICAP RQ inductively coupled plasma mass spectrometer (ICP-MS). The microwave digestion was performed using aqua regia-hydrofluoric acid with high chloric acid. The Hg content was determined using an AFS-8220 atomic fluorescence spectrophotometer. The digestion was conducted in a boiling water bath for 2 h after the addition of aqua regia. Mercury vapor was carried by the carrier gas into the atomizer for measurement. Standard quality assurance and quality control were strictly adhered to throughout the study. The recovery rate of heavy metals was between 70 and 125%, and the experimental results complied with the quality control standards.

### 2.3. Heavy metal pollution evaluation methods

#### 2.3.1. Nemerow index

##### 2.3.1.1. Single factor index.

A single pollutant can be quantitatively evaluated to determine the degree of exceedance of individual pollutants relative to background values and clarify pollution levels and main pollutants:


(1) $$P_{i}=C_{i}∕S_{i}$$

##### 2.3.1.2. Comprehensive index method.

An evaluation method based on a single factor pollution index can consider both the maximum and average values of individual pollutants. This method can comprehensively evaluate the overall pollution situation in the study area:


(2) $$P_{n}=\sqrt{\frac{{P_{\mathrm{imax}}}^{2}+{P_{ave}}^{2}}{2}}$$

where *P_i_* represents the single factor pollution index of pollutant *i* in soils; *C_i_* is the measured contamination value of pollutant *i*; *S_i_* denotes the assessment standard of pollutant *i* (21, 22); *P_n_* is the comprehensive pollution index for heavy metals in Inner Mongolia; *P_imax_* and *P_ave_* are the maximum and average value among single-factor pollution indices of each pollutant, respectively. In this study, *P_i_* was calculated for the five elements (Cd, Hg, Pb, As, and Cr).

#### 2.3.2. Ecological risk assessment

The potential ecological risk index method proposed by Lars (23) can comprehensively consider the ecotoxicity of pollutants and ecological environmental factors. This method can also reflect the combined effect of various pollutants on ecology. This index can be used to quantitatively analyze and predict potential ecological risks:


(3) $$RI=\overset{n}{\underset{i=1}{\sum }}E_{r}^{i}=\overset{n}{\underset{i=1}{\sum }}T_{i}\times P_{i}$$

where *RI* is the comprehensive potential ecological risk index, $E_{r}^{i}$ is the individual potential ecological risk index of heavy metal *i*, *T_i_* is the toxicity coefficient of heavy metal *i*, *P_i_* is the single factor index value for heavy metal *i*. The toxicity response coefficients of Cd, Hg, As, Pb, and Cr were 30, 40, 10, 5, and 2, respectively. The classification based on the heavy metal pollution assessment method is shown in Table 1.

**Table 1:** Heavy metal evaluation index.

Pollution index (*Pi/Pn*)		Potential ecological hazard index (Er/RI)		
		*E*_r_	RI	Evaluation
≤1	No pollution	<40	<150	Low risk
(1, 2)	Slight pollution	(40, 80)	(150, 300)	Moderate risk
(2, 3)	Light pollution	(80, 160)	(300, 600)	Considerable risk
(3, 5)	Moderate pollution	(160, 320)	(600, 1,200)	High risk
>5	Heavy pollution	>320	>1,200	Very high risk

#### 2.3.3. APCS-MLR receptor model

The APCS-MLR receptor model can be obtained by subtracting the principal component score of a zero concentration sample from the principal component scores from principal component analysis (24, 25). The calculation process includes three steps:

**Step (1)**: to standardize the raw data, introduce the zero concentration factor and calculate the APCS:


(4) $$Z_{ij}=\frac{C_{ij}-\bar{C_{j}}}{\sigma_{j}}$$

(5) $$Z_{j0}=\frac{0-\bar{C_{j}}}{\delta_{j}}=-\frac{\bar{C_{j}}}{\delta_{j}}$$

(6) $$\mathrm{APCS}=Z_{ik}-{\left(Z_{0}\right)}_{i}$$

where *Z_ij_* is the standardized value (dimensionless); *C_ij_* is the content of element *j* in the *i* sample (mg/kg); ${\bar{C}}_{J}$ and $\sigma_{j}$ are the mean and standard deviation of *C_ij_* (mg/kg).

**Step (2)**: multiple linear regression (MLR) analysis is performed using the APCS and heavy metal content as the independent and dependent variables, respectively. The contribution rate of individual pollutant sources is then calculated using regression coefficients.


(7) $$C_{i}=b_{i}+\overset{n}{\underset{m=1}{\sum }}\left(APCS_{im}\times a_{im}\right)$$

where *C_i_* is the content of the measured heavy metal element (mg/kg); *a_im_* represents the regression coefficient of source *m* on heavy metal element *i*; APCS_im_ represents the absolute principal component score of heavy metal element *i* for all samples; *b_i_* is a constant term in multiple linear regression; *APCS_im_ × a_im_* represents the contribution of source *m* to *C_i_* in the soil sample.

**Step (3)**: to avoid negative values affecting the results in the APCS-MLR modeling, the contribution rate of pollutant source *m* to heavy metal element *i* is calculated using the absolute value:

The contribution rate of a known pollutant source is expressed as


(8) $$PC_{im}=\frac{\left|a_{im}\times \bar{APCS_{im}}\right|}{\left|b_{i}\right|+\sum_{m=1}^{n}\left|a_{im}\times \bar{APCS_{im}}\right|}$$

The contribution rate of an unknown pollutant source is expressed as


(9) $$PC_{im}=\frac{\left|b_{i}\right|}{\left|b_{i}\right|+\sum_{m=1}^{n}\left|a_{im}\times \bar{APCS_{im}}\right|}$$

where *PC_im_* is the contribution rate for heavy metal element *i* and pollutant source *m* and $\bar{{\mathrm{APCS}}_{im}}$ represents the average of the absolute principal component scores of the entire sample for heavy metal element *i*.

### 2.4. Human health evaluation methods

#### 2.4.1. Heavy metal exposure models and parameters

Hazardous substances can enter human bodies through different transmission pathways. Health risk assessment is an effective method of determining the health risk level of various pollutants. It can evaluate the nature and possibility of the adverse effects on the human body caused by the chemical content of certain media in the current or future environment (26). Referring to the health risk model of the USEPA, the health risks of residents around the mining area were evaluated. The exposure pathways included oral ingestion, respiratory intake and skin contact (27–30). Human health risks were classified into noncarcinogenic and carcinogenic risks. This study evaluated the chronic non-carcinogenic risk of Cd, Hg, As, Pb and Cr and the carcinogenic risk of Cd, As, and Cr. The average daily intake of heavy metals by humans under three exposure pathways can be calculated using


(10) $$\mathrm{Oral ingestion:}\mathrm{AD}D_{\mathrm{ingest}}=\frac{\mathrm{Ci}\times \mathrm{IngR}\times CF\times EF\times ED}{BW\times AT}$$

(11) $$\mathrm{Inhalation:}\mathrm{AD}D_{\mathrm{inhala}}=\frac{\mathrm{Ci}\times \mathrm{InhR}\times EF\times ED}{PEF\times BW\times AT}$$

(12) $$\mathrm{Dermal contact:}\mathrm{AD}D_{\mathrm{dermal}}=\frac{\begin{array}{l} \mathrm{Ci}\times SA\times CF\times AF\times \\ \mathrm{ABS}\times EF\times ED \end{array}}{\;BW\times AT}$$

where *C_i_* is the content of heavy metal *i* in soils (mg/kg); other parameters refer to the USEPA health risk assessment standards and relevant research worldwide and are listed in Table 2.

**Table 2:** Parameters for soil heavy metal exposure risk.

Item	Parameters	Meaning	Unit	Reference value		Literature
				Adult	Child	
Common parameters	ED	Exposure years	a	24	6	(30)
	EF	Exposure frequency	d/a	350	350	(31–34)
	BW	Average weight	kg	56.8	15.9	(31–34)
	CF	Conversion factor	kg/mg^−1^	1 × 10^−6^	1 × 10^−6^	(31–34)
	AT (non-carcinogenic)	Average action time	d	ED × 365		(31–34)
	AT (carcinogenic)		d	70 × 365		(31–34)
Oral ingestion	IngR	Soil intake frequency	mg/d	100	200	(31–34)
Inhalation	InhR	Respiratory rate	m^3^/d	14.7	7.63	(31–34)
	PEF	Surface dust emission factor	m^3^/kg	1.36 × 10^9^	1.36 × 10^9^	(31–34)
Dermal contact	SA	Exposed skin surface area	cm^2^	5,075	2,448	(31–34)
	AF	Soil adsorption coefficient to skin	mg/cm	0.2	0.07	(31–34)
	ABS	Dermal absorption factor	dimensionless	0.001	0.001	(31–34)

##### 2.4.1.1. Non-carcinogenic risk

The chronic non-carcinogenic risk for human bodies exposed to soil heavy metals is calculated using:


(13) $${HQ}_{i}=\overset{5}{\underset{i=1}{\sum }}\frac{\mathrm{AD}D_{ij}}{RfD_{ij}}HI=\overset{5}{\underset{i=1}{\sum }}HQ_{i}$$

where *i* is the heavy metal species, *j* represents different exposure pathways, ADD (average daily dose) refers to the long-term daily exposure dose of heavy metals, RfD (reference dose) refers to the reference dose of non-carcinogenic heavy metals for daily intake, HQ represents the non-carcinogenic risk of potential toxic heavy metal elements in soils to human bodies and is obtained from the ratio of ADD of each heavy metal element to the corresponding RfD and HI is the total non-carcinogenic risk value for multiple heavy metals or one heavy metal under different exposure pathways.

##### 2.4.1.2. Carcinogenic risk

The human carcinogenic risk of heavy metals in soils is calculated as follows:


(14) $${CR}_{i}=\overset{3}{\underset{j=1}{\sum }}\mathrm{AD}I_{ij}\times {SF}_{ij}TCR=\overset{3}{\underset{i=1}{\sum }}CR_{i}$$

where ADI (carcinogenic heavy metals) is the long-term daily average exposure dose of heavy metals [mg/(kg·d)]; SF (slope factor) refers to the slope factor of carcinogenic heavy metals; CR is the carcinogenic risk caused by the potential toxic heavy metal elements in soils to human bodies and can be obtained by multiplying ADD of each heavy metal element by its corresponding SF; TCR represents the total carcinogenic risk of multiple heavy metals or a single heavy metal under multiple exposure pathways (dimensionless). The values of RfD and SF are shown in Table 3 (30–34).

**Table 3:** RfD and SF values of heavy metals under different exposure pathways.

Element	RfD /mg·(kg·d)^−1^			SF /(kg·d)·mg^−1^		
	Oral ingestion	Inhalation	Dermal contact	Oral ingestion	Inhalation	Dermal contact
Cd	0.001	0.001	0.00001	6.1	6.3	6.1
Hg	0.0003	0. 0003	0.000021	-	-	-
As	0.0003	0.0003	0.0012	1.5	15.1	3.66
Pb	0.0035	0.0035	0.00052	-	-	-
Cr	0.003	0.000029	0.00006	0.5	42	20

Considering the actual situation of the area and relevant USEPA regulations, the health risk model evaluation classification values are shown in Table 4.

**Table 4:** Health risk model evaluation criteria.

Range	Risk level
HQ/HI < 1	Negligible non-carcinogenic risk
HQ/HI > 1	Non-carcinogenic risk exists and cannot be ignored
CR/TCR ≤ 1.00E-06	Carcinogenic risk exists and can be neglected
1.00E-06 < CR/TCR ≤ 1.00E-04	Carcinogenic risk exists and is acceptable.
CR/TCR > 1.00E-04	Carcinogenic risk exists and is unacceptable.

### 2.5. Statistical analysis and geospatial distribution

The SPSS 21.0 software was used for descriptive statistics, Pearson correlation analysis, principal component analysis and operation of the APCS-MLR receptor model on heavy metal data from sampling. The ArcMap 10.8 software was used to draw a schematic diagram of the sampling points and a distribution map of heavy metal pollution. Origin 2019 was used to illustrate the relevant statistical results.

## 3. Results

### 3.1. Soil characterization

The sample size at the sampling points was *n* = 190. The pH value of agricultural soils in the study area ranged from 4.5 to 8.24, with an average of 6.20, indicating an overall acidic soil. According to Table 5, except for Cd, all other elements exceed the soil background values of Guizhou Province, with Hg having the highest exceedance (89.47%). The exceedance of Hg, As, and Pb was all higher than 50%. In contrast, the exceedance of Cd and Cr was relatively low. The coefficients of variation for individual elements were as follows: Hg (0.55) > As (0.65) > Cd (0.61) > Pb (0.31) > Cr (0.29). Except for the element Cr, the coefficients of variation for the other elements were all greater than 0.3, indicating moderate to high variability. The coefficients of variation for Cd, Hg and As fell between 0.5 and 1, indicating a higher degree of variability. This suggests that the variations in the Cd, Hg and As content in the study area were most significantly influenced by anthropogenic activities.

**Table 5:** Descriptive statistics of soil heavy metals.

Statistics	Element				
	Cd	Hg	As	Pb	Cr
Min (mg/kg)	0.10	0.05	2.16	13.20	24.20
Max (mg/kg)	1.46	0.68	79.40	74.00	199.00
Mean (mg/kg)	0.44	0.24	26.16	39.16	94.56
Skewness	1.73	1.27	1.13	0.54	0.63
Kurtosis	3.22	1.16	0.69	0.15	0.99
Coefficient of variation	0.61	0.55	0.65	0.31	0.29
Exceedance (%)	15.26	89.47	54.74	58.95	43.16
Average background of Guizhou Province	0.66	0.11	20	35.2	95.90

### 3.2. Pollution assessment results

The assessment results of heavy metal pollution in the cropland of the study area are shown in Table 6. The single factor pollution index method indicates that Hg was the main pollutant in the study area. The averages of the one-way pollution index of various elements were Hg (2.15) > As (1.31) > Pb (1.11) > Cr (0.99) > Cd (0.67). Except for the Cd element, the heavy metal contents at the sampling points lay within the clean range. However, the contents of other elements exceeded the background value to some extent, with As, Pb and Cr exhibiting slight exceedance. The mean values of the potential ecological pollution indices for individual heavy metals were Hg (86.01) > Cd (20.03) > As (13.08) > Pb (5.56) > Cr (1.97). The mean value of the comprehensive potential ecological risk index for the entire area was 126.65. This indicates that the Hg content in the study area will pose potential moderate ecological risks, and the other four types of elements will pose slight ecological risks. The comprehensive potential ecological risk of the entire area was classified as slight.

**Table 6:** Calculated pollution indices due to heavy metal concentrations.

Element	*Pi*			*Er*		
	Average	Min	Max	Average	Min	Max
Cd	0.67	0.15	2.21	20.03	4.64	66.36
Hg	2.15	0.44	6.16	86.01	17.45	246.55
As	1.31	0.11	3.97	13.08	1.08	39.70
Pb	1.11	0.38	2.10	5.56	1.88	10.51
Cr	0.99	0.25	2.08	1.97	0.50	4.15
*Pn*	2.50					
*RI*	126.65					

### 3.3. Pollution source analysis

#### 3.3.1. Pearson correlation matrix

In order to understand the correlation between various heavy metals in the soil samples, Pearson’s correlation analysis was conducted on the raw data from the sampling points. Table 7 shows that the correlation coefficients between Hg-As-Cr and As-Pb-Cr, two groups of combined elements, were above 0.5. They all exhibited a significant positive correlation (*p* < 0.01) (35). This indicates that Hg, As, Pb, and Cr had similar pollution sources.

**Table 7:** Correlation analysis of heavy metals.

Element	Cd	Hg	As	Pb	Cr
Cd	1				
Hg	0.096	1			
As	−0.083	0.591^**^	1		
Pb	0.225^**^	0. 454^**^	0.657^**^	1	
Cr	0.122	0.629^**^	0.550^**^	0.439^**^	1

** Indicates the significance at *p* < 0.01.

#### 3.3.2. Source apportionment using the APCS-MLR model

Principal component analysis was conducted using the SPSS software. The results of the Bartlett’s spherical test and the KMO measure were 0.000 and 0.65, respectively. This indicates that it was suitable for principal component analysis. Combining the correlation analysis results, the standardized factors of the two extracted principal components were subjected to orthogonal rotation. The cumulative contribution rate reached 72.528%, which can represent most of the information on the research object.

#### 3.3.3. APCS-MLR model

Through principal component analysis, the two principal component scores were converted into APCS. Then, in order to obtain the contribution rates of different pollution sources, the APCS and heavy metal content were used as independent and dependent variables, respectively, to conduct multiple linear regression analysis. The APCS-MLR receptor model results for individual heavy metal elements are shown in Table 8.

**Table 8:** Linear regression models.

Receptor model	R^2^	Significance
CCd = −0.068 + 0.021APCS1 + 0.26APCS2	0.953	0.000
CHg = −0.1 + 0.105APCS1 + 0.013APCS2	0.648	0.000
CAs = −6.977 + 14.377APCS1-5.78APCS2	0.823	0.000
CPb = 10.786 + 8.821APCS1 + 1.162APCS2	0.537	0.000
CCr = 21.76 + 21.608APCS1 + 4.753APCS2	0.651	0.000

### 3.4. Health risk assessment

#### 3.4.1. Non-carcinogenic risk

Table 9 shows that the HI and HQ values for both adults and children were less than 1. Therefore, the exposure to heavy metals in the study area posed a negligible non-carcinogenic risk to the human body within the acceptable range.

**Table 9:** Non–carcinogenic risks to humans from soil heavy metals.

Element	HQ_Adults_				HQ_Children_			
	Mean	Median	Min	Max	Mean	Median	Min	Max
Cd	5.14E-04	4.32E-04	1.19E-04	1.70E-03	4.95E-04	4.15E-04	1.14E-04	1.64E-03
Hg	1.52E-03	1.22E-03	1.59E-04	4.37 E-03	9.63E-03	7.77E-03	1.95E-03	2.76E-02
As	5.06E-02	4.24E-02	3.87E-05	1.54E-01	9.02E-02	7.62E-02	7.45E-03	2.74E-01
Pb	2.01E-02	1.91E-02	6.61E-05	3.81E-02	1.36E-01	1.28E-01	4.58E-02	2.56E-01
Cr	2.77E-02	2.68E-02	7.09E-03	5.83E-02	3.41E-02	3.29E-02	8.72E-03	7.17E-02
HI	9.62E-02				2.70E-01			

#### 3.4.2. Carcinogenic risk

Table 10 shows that the CR and TCR values for both adults and children were less than 1.00E-04. Therefore, the exposure to heavy metals in the study area posed a negligible carcinogenic risk to the human body, within the acceptable range.

**Table 10:** Carcinogenic risks to humans from soil heavy metals.

Element	CR_Adults_				CR_Children_			
	Mean	Median	Min	Max	Mean	Median	Min	Max
Cd	1.57E-06	1.32E-06	3.64E-07	5.21E-06	2.78E-06	2.34E-06	6.44E-07	9.22E-06
As	2.33E-05	1.97E-05	1.92E-06	7.07E-05	4.07E-05	3.44E-05	3.36E-06	1.23E-04
Cr	3.87E-05	3.74E-05	9.91E-06	8.15E-05	5.07E-05	4.90E-05	1.30E-05	1.07E-04
TCR	6.36E-05				9.41E-05			

## 4. Discussion

### 4.1. Analysis of heavy metal pollution

In the preliminary analysis, this study performed a descriptive analysis on the heavy metal contents in the soil samples (Table 5). The results show that the variation coefficients of each element were in the order of Hg > As > Cd > Pb > Cr. Except for the element Cr, the variation coefficients of all other elements exceeded 0.3, indicating medium or high variations. The variation coefficients of Cd, Hg and As were between 0.5 and 1, indicating a high degree of variation (36). This suggests that the variations in the Cd, Hg and As contents in the study area were most significantly affected by human activities.

**Figure 2:**
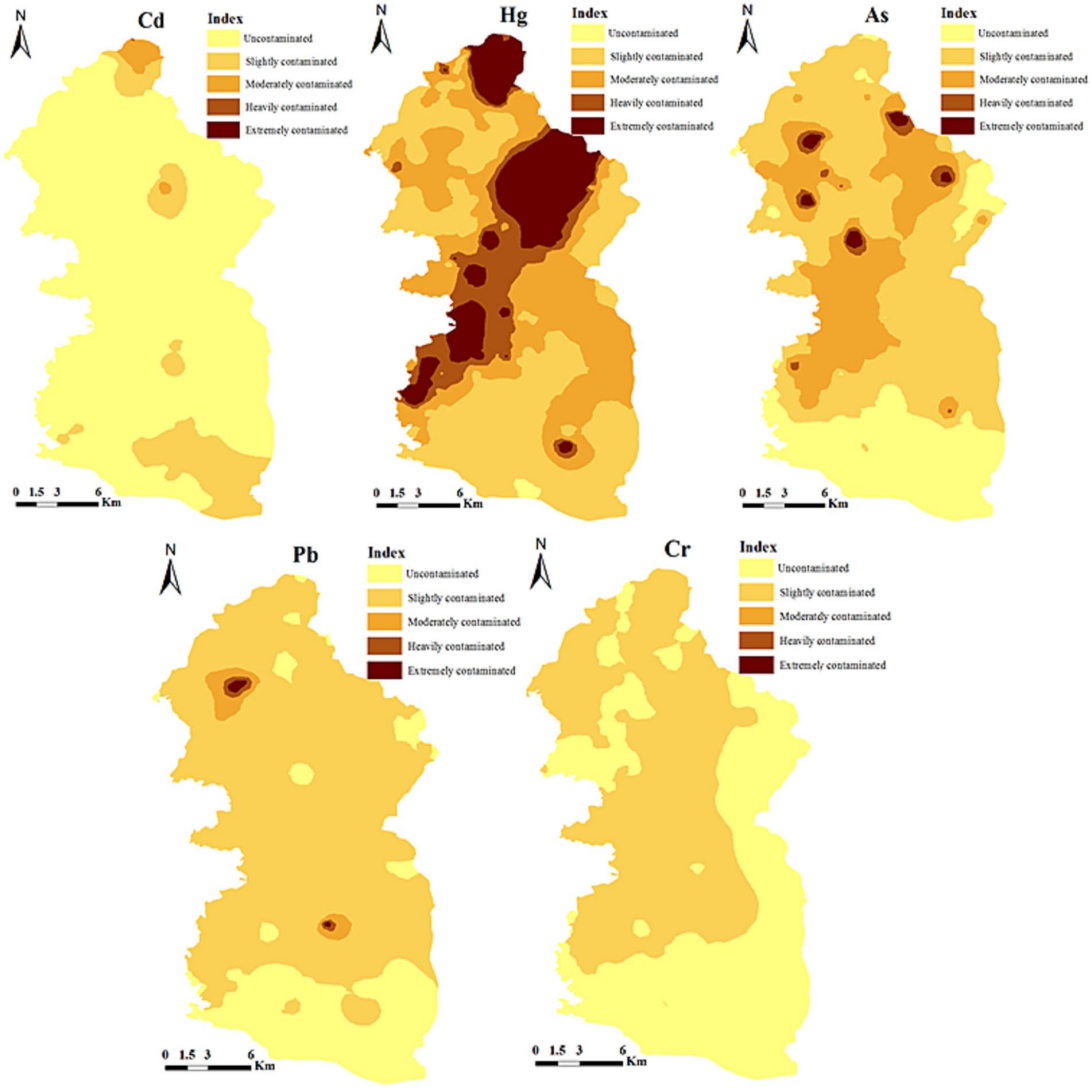
Single factor pollution distribution maps of heavy metals.

The single factor pollution index of individual elements was interpolated by inverse distance weighting to depict the pollution status of each element (Figure 2). Figure 2 shows that there were high pollution areas with point-like distributions for Cd, Hg, As and Pb in the pollution status map of individual elements. This indicates that these four elements were enriched due to human activities. In the study area, 84.74% of the soil samples were clean in Cd, and 15.26% exceeded the Cd standard limit and were in a polluted state with point-like distributions. Most of the soil samples did not exceed the standard limit of Cd. Regarding Hg, 89.47% of the samples were in a polluted state, among which 5.26% were severely polluted. High pollution points exhibited patchy distributions, indicating the largest influence of human activities. In terms of Pb, 58.95% of the sampling points were categorized as “slightly polluted,” most of which were distributed in the upper reaches of the river. In terms of As, 45.26% of the sampling points were in a clean state, most of which were slightly polluted. The clean sampling points accounted for 56.84% with respect to Cr, with moderate coefficients of variation. This indicated fewer influences from anthropogenic pollution. The average pollution index for the entire area was 2.50, indicating mild pollution. Hg was the biggest contributing factor to the comprehensive pollution level in this area, with a contribution rate of 36.90%.

According to the contribution rates of each element to the slight ecological risk level in the region (Hg, 67.91%; Cd, 15.81%; As, 10.33%; Pb, 4.39%; Cr, 1.56%) in Table 6, Hg was the main factor causing the potential ecological risk in the region. Therefore, relevant departments should pay attention to the potential slight ecological risk that the Hg content may pose to the area.

**Figure 3:**
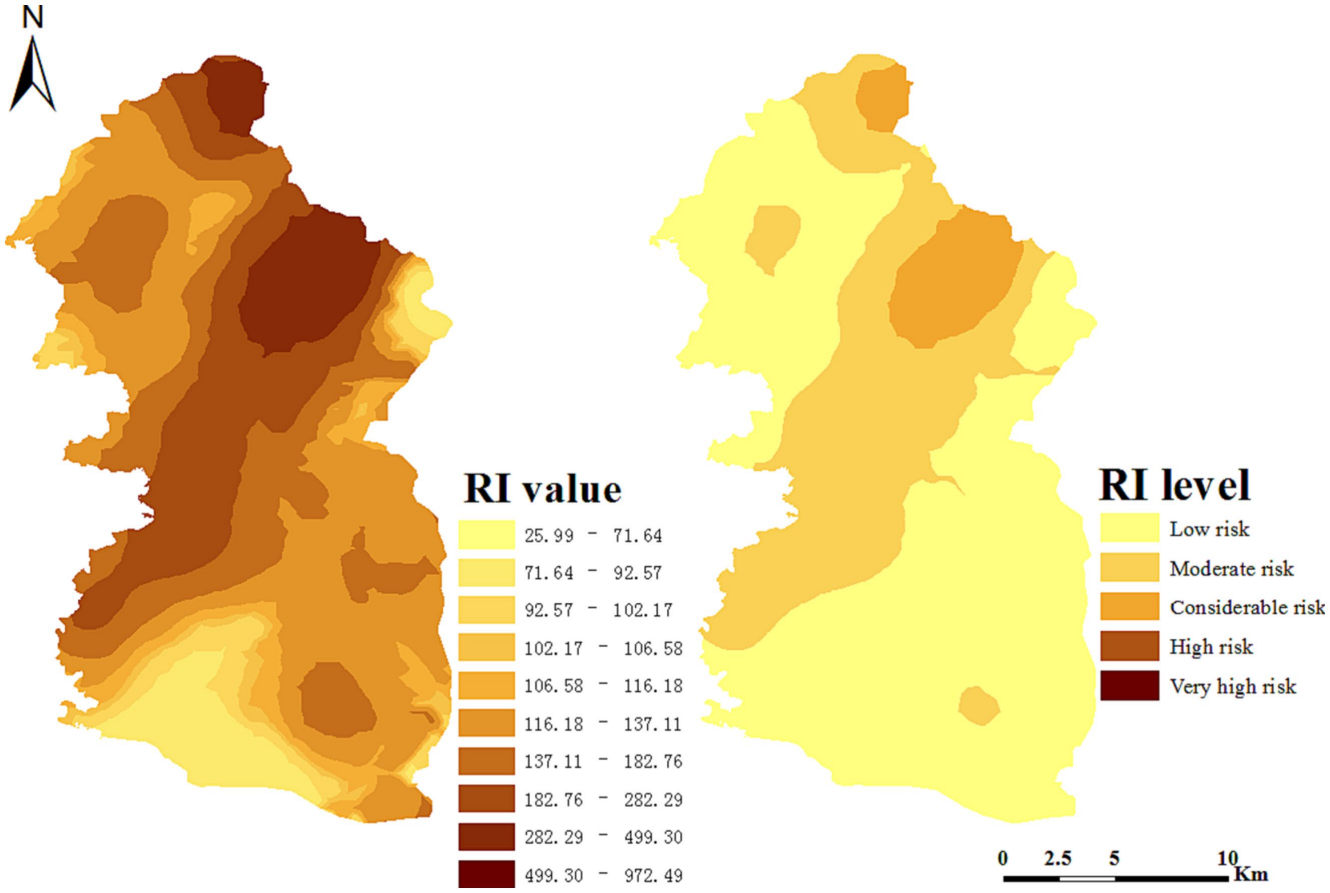
Maps of potential ecological risks.

The spatial distribution of potential ecological risks in the study area is shown in Figure 3. According to the RI value, there were patch-shaped areas with high potential ecological risks, most of which were unevenly distributed. This indicates that the soil was contaminated and enriched by human activities. The RI level analysis shows that the proportion of areas with slight pollution in the entire area was the largest. In contrast, the proportion of areas with very serious potential ecological risks was the smallest. The area with the largest mining area was the most severely contaminated in the entire area, and the spatial distribution showed a trend of gradually increasing potential ecological risks from southwest to northeast. The investigation and analysis also show that the areas with severe pollution were the mining areas and regions with developed transportation. Thus, it can be concluded that industrial and mining activities are one of the causes of the spatial distribution of potential ecological risks in this region.

### 4.2. Analysis of heavy metal pollution sources

#### 4.2.1. Pearson correlation matrix

The correlation analysis in Table 7 shows that stronger correlations indicated higher similarity of pollution sources. The correlation between Cd and other heavy metals was insignificant, suggesting that Cd may result from other pollution sources. The correlation results of this study were similar to those of previous studies on the sources of heavy metals in typical farmland soils in Huibu Town, Changshan County, West Zhejiang. There were two types of pollution sources, both due to frequent industrial and mining activities in the previous and present study areas. However, the present study area is distinctive because it has many roads and railways, indicating that the pollution sources were more likely to come from industrial and mining activities.

#### 4.2.2. PCA-APCS-MLR

The Hongfeng Lake watershed is located in Guizhou Province, which is rich in mineral resources. The surrounding area of the Hongfeng Lake watershed is primarily the alumina ore mining development zone in the Qiannan region (37). Many industrial and mining enterprises release heavy metals into the environment through exhaust gas, wastewater and waste residues. Mineral exploitation can lead to the accumulation and pollution of heavy metal elements in the soil (38, 39). Heavy metal pollution has been identified in the sediments of the Hongfeng Lake watershed, with the pollution level of each element as follows: Hg > Pb > As, and the potential ecological risk pollution level was moderate. The main pollution source for the primary pollutant, Hg, is the wastewater pollution generated by chemical plants and industrial enterprises around the lake (40). The farmland soil in the watershed is part of the aquatic ecosystem, and the farmland soil conditions interact with water quality. Through agricultural activities such as ecological migration, planting and irrigation, the deposition and pollution of heavy metals can occur in the farmland soil.

Therefore, based on previous research results, the exploration results of the sources of heavy metal pollution in the surrounding farmland of the watershed are as follows. The principal component analysis (Table 11) shows that the cumulative variance contribution rate of PC1 was 50.402%. The variables with the highest factor loadings on PC1 were Hg, As, Pb, and Cr, with loadings of 0.802, 0.842, 0.730, and 0.790, respectively. The evaluation and analysis of heavy metal pollution show that the Hg, As, and Pb contents were higher than the background values of soils. Hg had a slight cumulative pollution, and they all had medium-high variability. This indicates that these heavy metals were greatly affected by human activities and had similar pollution sources. Moreover, the pollution distribution map (Figure 2) of individual elements shows that these three heavy metals had similar local high-pollution distributions, mostly located in areas with intensive mining activities. Previous studies have shown that mining activities in mining areas may aggravate the contamination of heavy metals such as Hg, As and Pb in the region. During mineral mining, rock blasting, grinding and ore beneficiation can cause the migration of As and Pb fine powders to surrounding soils, significantly increasing the As and Pb contents in the soil (35, 41, 42). Artisanal smelting and tailings are the main Hg sources. These metal elements pollute the soil through atmospheric deposition, weathering and surface runoff, and exhaust emissions from trucks transporting minerals contribute to the Pb pollution in farmland soils (3, 43, 44). Cr did not exceed the background value, with a low coefficient of variation, and no cumulative pollution occurred. Thus, Cr was less affected by human activities. Combined with previous studies, Cr may be influenced by natural factors such as parent materials and bedrocks. Therefore, the pollution sources of the first principal component include industrial and mining activities and natural sources.

**Table 11:** Rotational component matrix of heavy metals.

Component matrix after rotation		
Element	Component	
	PC1	PC2
Cd	0.080	0.973
Hg	0.802	0.097
As	0.842	−0.339
Pb	0.730	0.096
Cr	0.790	0.174
Eigenvalue	2.520	1.106
Cumulative variance contribution rate (%)	50.402	72.528

The cumulative contribution rate of PC2 was 72.528%. The main loading factor was Cd, with a coefficient of 0.973. Based on the analysis of heavy metal contents and pollution status, the Cd content in the study area did not exceed the standard limit. This indicated a clean status. The coefficient of variation was 0.61, indicating medium-high variations and more significant influences of anthropogenic factors. Studies have also shown that fertilizers contain a large amount of Cd, and pesticides and fertilizers can cause Cd pollution (45, 46). This indicates that the pollution sources of the second principal component are agricultural sources.

**Figure 4:**
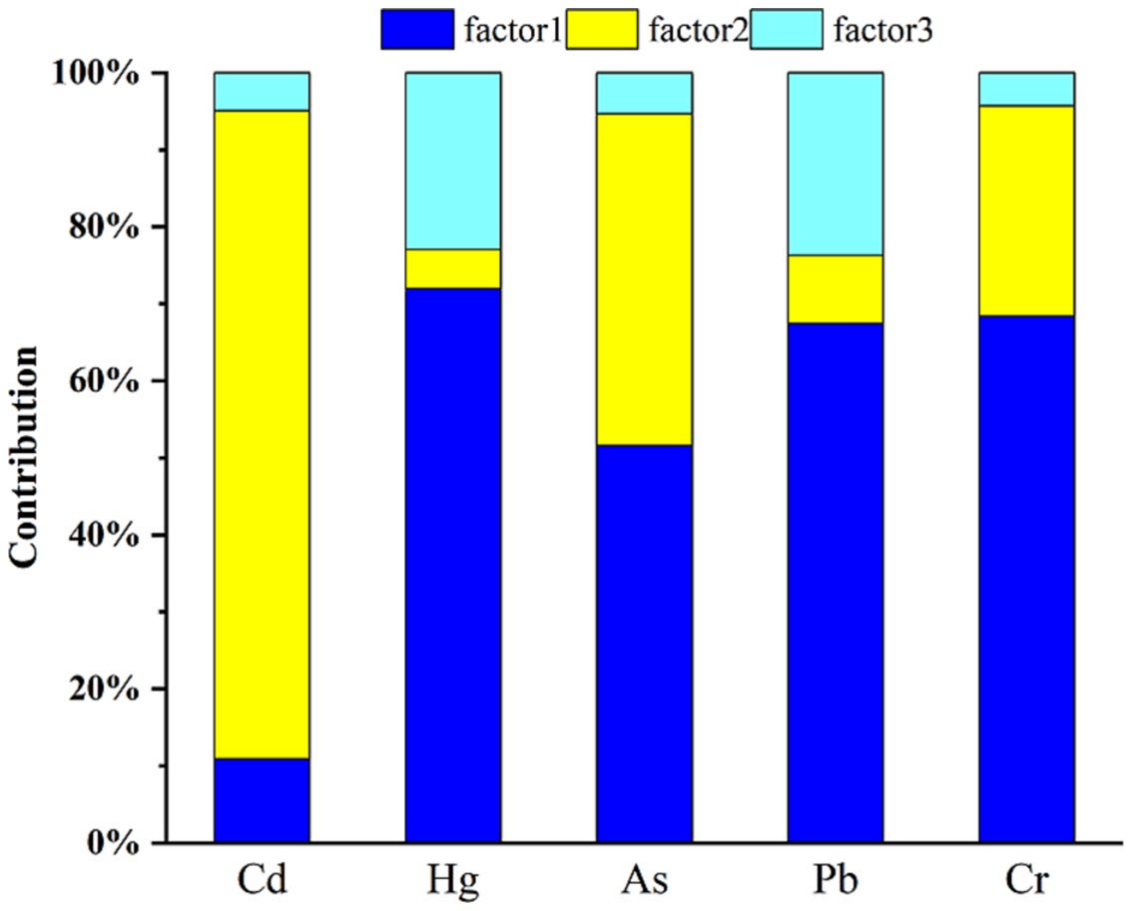
Contributions of heavy metal pollution sources.

The contribution rates of heavy metals based on the APCS-MLR model combined with principal component analysis are shown in Figure 4. Factor 1 was attributed to industrial and mining activities and parent material pollution, including Hg, As, Pb, and Cr, with contribution rates of 71.99, 51.57, 67.39, and 68.36%, respectively. Factor 2 was attributed to agricultural pollution, mainly due to Cd, with a contribution of 84.12%. It should be noted that As and Cr accounted for a relatively large proportion in Factor 2, with contribution rates of 43.11 and 27.31%, respectively. Factor 3 was attributed to unknown sources of heavy metal pollution, including Cd (4.95%), Hg (22.98%), As (5.32%), Pb (23.73%), and Cr (4.32%).

### 4.3. Health risk of heavy metals in locals

Tables 9, 10 show that the HI and HQ values for adults and children were both less than 1, and the TCR and CR values were both below 1.00E-04. This indicates that the health risks posed by heavy metals to the human body were within an acceptable range. Among the non-carcinogenic risks, the mean HQ values of As for adults (5.06E-02) and children (9.02E-02) were the highest. In contrast, the mean HQ values of Cd for adults (5.14E-04) and children (4.95E-04) were the lowest. In terms of carcinogenic risk, the mean CR values of Cr for adults (3.87E-05) and children (5.07E-05) were the highest. In contrast, the mean CR values of Cd for adults (1.57E-06) and children (2.78E-06) were the lowest. Therefore, As was the main contributor to non-carcinogenic risks in the human body, and Cr was the main contributor to carcinogenic risks. Cd posed the lowest health risk to the human body. It is particularly noteworthy that the maximum values of As (1.23E-04) and Cr (1.07E-04) in carcinogenic risks exceeded 1.00E-04, which may pose significant carcinogenic risks to the human body. This indicates that the human health risks in the study area are generally acceptable. A few high-value points that pose significant carcinogenic risks cannot be ignored.

**Figure 5:**
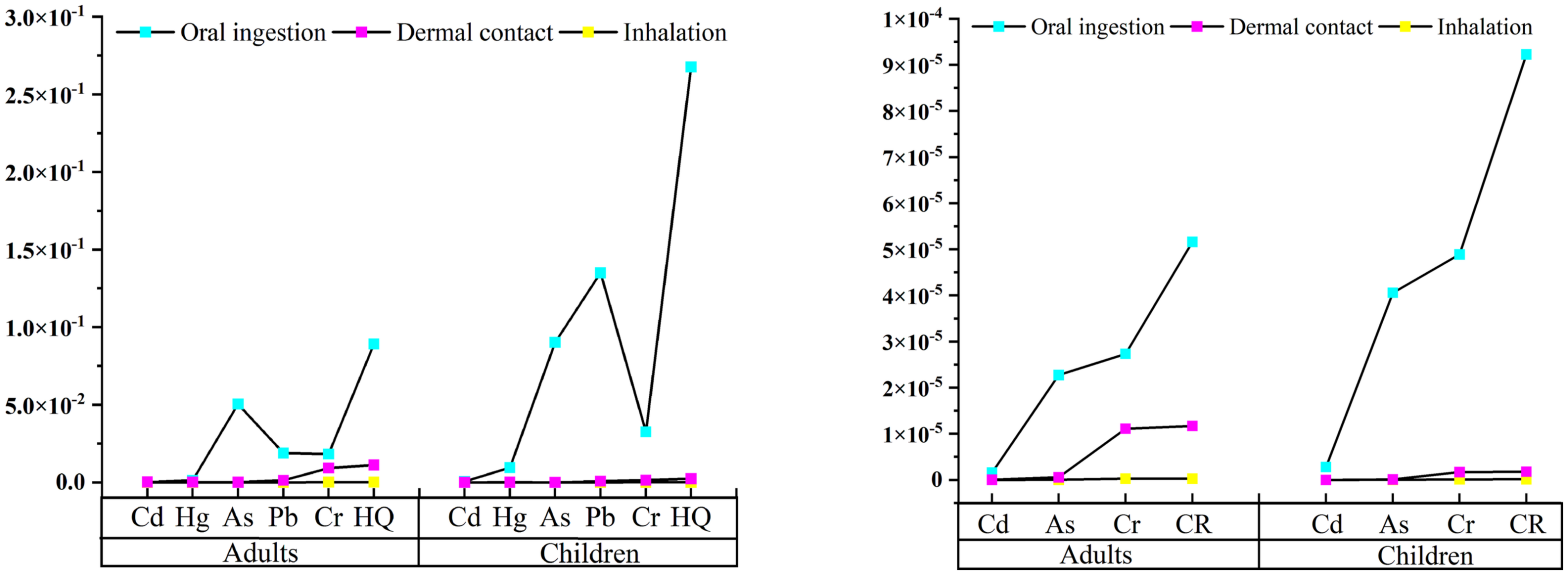
Health risk values under different pathways.

The average amount of heavy metals ingested by the human body through different exposure pathways is shown in Figure 5.

Figure 5 shows that the amount of heavy metals in each ingestion pathway is in the following order: oral ingestion > skin absorption > inhalation. Oral ingestion is the main pathway for heavy metals to pose health risks to the human body, consistent with previous research. Children have certain physiological and behavioral differences compared to adults, such as weaker detoxification ability, finger-sucking behavior, greater skin exposure area and higher vulnerability (30–34, 47). Therefore, we should pay attention to children’s exposure pathways to heavy metal. Health education and publicity should be strengthened to enhance their hygiene awareness. The total non-carcinogenic risk value (HI) in the study area was less than 1. The total carcinogenic risk value (TCR) was between 1.00E-06 and 1.00E-04. This indicates that the health risks of soil heavy metals to the human body in this area are within an acceptable range. However, potential health hazards posed by the primary elements causing health risks should be prevented.

## 5. Conclusion

The development of the mining industry and agricultural activities in the upstream of Hongfeng Lake aggravated the heavy metal pollution in agricultural soil. The development of the mining industry resulted in high Hg accumulation in the soil. Multiple high pollution points were distributed in areas with the mining industry and road traffic. The overall pollution level of the study area was classified as mild, with Hg being the primary pollutant. As and Pb were secondary pollutants and were classified as slight pollution. Cd and Cr were in a clean state, without pollution. The overall potential ecological risk level in the entire area was considered slight. Hg was the main factor causing potential ecological risks in this region.

Descriptive data shows that the coefficients of variation for Cd, Hg and As ranged between 0.5 and 1, indicating a high degree of variability. They were more greatly influenced by human activities. The pollution source analysis using PCA combined with the APCS-MLR model reveals that the main pollution sources for Hg, As, Pb, and Cr were related to industrial and mining activities and natural sources. The main source of Cd pollution was agricultural activities. Agricultural sources also contributed significantly to As and Cr. Therefore, in future development, attention should be paid to the combined water and soil pollution.

Heavy metals did not pose a non-carcinogenic risk to human health but had an acceptable carcinogenic risk. As and Cr were the main non-carcinogenic and carcinogenic factors, respectively. Children had a higher hazard index (HI) and target cancer risk (TCR) values than adults due to physiological and behavioral differences. For example, children are more susceptible to the health risks of heavy metals. This is because they tend to suck their fingers, come into contact with substances and have lower detoxification abilities.

## 6. Limitations and future perspectives

This study highlights the impact of heavy metal contents in agricultural soils on human health, with an emphasis on cropland soils. It does not cover all soil types. For a more in-depth exploration, other soil types could be included in future research. There are multiple variables involved in heavy metal pollution sources and human health risk assessment. For example, there is a lack of detailed pollution inventories for comparison of pollution sources. Human health risk studies are based on exposure levels to heavy metals in soils. The subjectivity of individuals is overlooked. These can limit the variability of the study. Exploring pollution sources and research methods for human health risk assessment will also involve studying more advanced and timely approaches. In future studies, other research methods will be incorporated for comparison to better support the conclusions and provide a more comprehensive understanding.

## Data availability statement

The original contributions presented in the study are included in the article/Supplementary material, further inquiries can be directed to the corresponding author.

## Author contributions

WC: Conceptualization, Data curation, Investigation, Methodology, Project administration, Resources, Software, Supervision, Validation, Writing – original draft, Writing – review & editing. YM: Conceptualization, Data curation, Investigation, Methodology, Project administration, Software, Validation, Visualization, Writing – original draft, Writing – review & editing. SL: Data curation, Formal analysis, Funding acquisition, Investigation, Methodology, Resources, Writing – review & editing. XZ: Conceptualization, Methodology, Resources, Software, Visualization, Writing – review & editing.
